# Report of a Case of Lithotomy

**Published:** 1880-01

**Authors:** A. E. Baldwin


### ARTICLE V.

### REPORT OF A CASE OF LITHOTOMY. By A. E. BALDWIN, M.D. Read before the Military Tract Medical Society.

I was consulted June 14, 1879, by Frank Powell, a young man aged 19, in regard to his ailments. Said he had not been *well* for many years; had always been troubled the same as now, only not so severely as for the past three weeks, during which time he has been suffering severely from pain in the lower part of the abdomen, always dull and heavy in character.

Has lost flesh rapidly for the last three weeks, is now quite emaciated and anæmic.

Upon inquiry, I found he had to urinate very frequently, many times wanting to, but unable to do so. Sometimes while urinating urine would stop suddenly; felt no itching about penis, neither were the testicles retracted.

I told him I suspected vesical calculus, and suggested the use of the sound; after urging, he consented, and introducing the sound I found the prostate much enlarged. As soon as the sound entered the bladder it struck the stone with so decided a *click* that the patient heard it. The stone was apparently of pretty large size, and I advised him to have it removed. By request of the young man's guardian, I took him to Dr. J. Todd, of Galva, who fully confirmed my diagnosis. I gave the patient opiates and mild laxatives, and fixed upon June 24th, 1 P. M., for operation. As he lived some distance in the country (8 miles), arrangements were made to have the operation performed and patient cared for here at the Toulon House. Advised patient to keep as quiet as possible, and I came to town the day before hand.

June 23d.—Patient came to town; gave him at bedtime, ℥vi. (24 c. c.) of castor oil, which operated well in early morning. Ordered him an injection at 10 A. M., followed by one-sixth grain morphia.

June 24th, P. M.—Having arranged a table and covered it—as per authors—I proceeded to the operation. My assistants were Drs. D. H. Alvis, gave ether; J. F. Todd, staff holder; E. R.

Boardman and J. B. Ingals, held legs in position. Drs. R. O. Phillips and W. T. Hall were present by invitation, to witness the operation. About 2:20 commenced giving the anæsthetic and at 2:30, having placed my instruments in a chair to reach them handily, I shaved the perinæum; then made my first incision, beginning just to the left of the raphe, about $1\frac{1}{4}$ inches in front of the anus, carried it obliquely outwards and backwards, terminating it a little posterior to and about midway from the tuberosity of ischium and anus.

Second incision was made with forefinger of left hand in anus and beginning about $\frac{1}{2}$ inch farther back than first—cut quite deeply—was compelled to ligate transversalis perinei artery. Then introduced fore-finger of left hand into the wound, sought for the groove in the staff; finding it, I traced the groove as far back as I could to be sure and avoid the bulb of urethra and artery; then, pressing the nail well into groove and finger in front of rectum, I cut into the groove. Then, placing the probe-pointed knife in groove, and, giving it the same angle as the wound, I grasped the staff, pulled it well up under the arch and pushed the knife forward into the bladder.

Introduced finger into bladder over staff, then withdrew staff and felt for the stone; found it lying, seemingly, in a pouch just back of prostrate gland. Introduced forceps over finger, and, with the aid of the finger in the bladder, grasped the stone. The spread of the forceps showing that we had a very large stone, after repeated efforts of dilatation and traction, found I could not get the stone out of present opening nor crush the stone.

I then cut across the raphe, converting it into a "medio-lateral" operation, and, not succeeding then, was compelled to convert it into a "bi-lateral" by making a similar incision on right side as that on the left, not quite as long externally, and, after repeated efforts, dilating wound and turning the stone, I succeeded in removing it. I then washed out the bladder with tepid water, and, as there was some oozing from the wound, washed out the wound with ice water, and had patient conveyed to bed—previously prepared and well covered.

The stone was oblong, rough and warty, about 6.5 ctm. in length, 5 ctm. in breadth and 3.3 ctm. thick; weighed about 3 oz. Patient lost 3 to 5 ounces of blood.

7 p. m.—Found patient in considerable pain and a little feverish; ordered him pills of morph. ($\frac{1}{4}$ gr.) as often as necessary to keep him easy. Washed wound and dressed it with carbolized sol. (1 to 60) after anointing buttocks with olive oil to prevent irritation.

June 25th a. m.—Pulse, 92; temp., 37 2-5°. Says he is very comfortable. Treatment continued, with patient taking milk 60. every two hours.

P. m.—Pulse, 88; temp., 37 4-5°; resting well; has not had morph. for several hours.

June 26th.—Was called up during the night to draw off the urine, which I did by introducing catheter through the penis without any serious pain; provoked a little flow of blood from wound.

7 a. m.—Pulse, 90; temp., 37 1-5°; patient prospering nicely, and everything favorable.

1 p. m.—Pulse, 110; temp., 40°; skin hot; feels well; only lame and head aches. Ordered cinchonidia sulph. 0.4 every 1$\frac{1}{2}$ hours.

3 p. m.—Pulse, 122; temp., 41.5°; skin very hot and dry; abdomen tender and swollen; has had chills twice. Tongue coated and dry; nervous, restless and delirious; continued treatment with addition of

| | | |
|---|---|---|
| ℞ Tr. aconit. rad.................................... | | 26 |
| Pot. chlorate.......................................... | | 66 |
| Tr. opii.................................................. | 1 | 33 |

M. Sig. In syrup every hour as long as necessary.

7 p. m.—Pulse, 110; temp., 40°; some improvement.

11 p. m.—Pulse, 85; temp., 39°; skin somewhat hot but moist; says he is comfortable, but tired. Throat somewhat sore and dry; thinks he can sleep.

June 27th—6 a. m.—Pulse, 85; temp., 37.6°; patient rested well; throat feels better; skin and tongue moist; abdomen better, only complains of being tired.

11 a. m.—Pulse, 84; temp., 36.7°; skin moist; has eaten crackers in milk.

6 p. m.—Pulse, 85; temp., 37.9°.

10 p. m.—Pulse, 82; temp., 37.3°.

June 28th—9 a. m.—Rested well during night; urine comes through wound again. Pulse, 80; temp., 37.3°; tongue cleaning; not much swelling about wound.

2 p. m.—About same; ate oysters to-day.

9:30 p. m.—Same. Ate some chicken.

June 30th.—Gave injections and removed a large amount of fæcal matter.

July 3d.—Water began coming through urethra again; wound healing very fast; continued to dress with carbolic wash. Continued to improve and the 11th of July patient was discharged, wound all healed. Has gained eight pounds since operation, (had lost twenty-six in the five weeks previous to operation.)

July 19th.—Heard patient was doing light work; gaining fast.

Dec. 15th.—Heard from him a few days ago. Says he is perfectly well; weighs more than ever before, and can do as much work as any man in the country.

---

THE AUDIPHONE.—Frequent inquiries regarding this much-vaunted invention induce me to state that Messrs. Sharp & Smith of this city have been carefully testing the audiphone for several weeks, but thus far they have not found patients benefited by it. Out of one hundred and fifty successive cases of deafness, in which they have given it a trial, they report that not a single one has noticed any benefit in its use.—E. FLETCHER INGALS, M.D.

PROF. SARAH HACKETT STEVENSON having been obliged to ask for a year's leave of absence from the Chair of Physiology at the Woman's College, Prof. F. L. Wadsworth has kindly consented to supply her place for the present session, and we may add that his course of instruction is giving very great satisfaction to both class and faculty.

